# Effect of Curing Condition on Resistance to Chloride Ingress in Concrete Using Ground Granulated Blast Furnace Slag

**DOI:** 10.3390/ma12193233

**Published:** 2019-10-02

**Authors:** JangHyun Park, Cheol Park, SungHyung Joh, HanSeung Lee

**Affiliations:** 1 Architectural Engineering, Hanyang University, Seoul 04763, Korea; narus7@hanyang.ac.kr; 2 Concrete Research Office, Technology Research Center, Ssangyong Cement Industrial Co., Ltd., Daejeon 34115, Korea; chpark@ssyc.co.kr (C.P.); kurisuma@ssyc.co.kr (S.J.); 3 School of Architecture and Architectural Engineering, Hanyang University, ERICA, Ansan 15588, Korea

**Keywords:** concrete, salt attack, ground granulated blast furnace slag, curing condition, diffusion coefficient, electrochemical impedance spectroscopy

## Abstract

Changes in the salt attack resistance of concrete using ground granulated blast furnace slag (GGBFS) were examined based on different curing conditions. These conditions were divided into air and underwater curing. Three concrete mixes with GGBFS replacement ratios of 0% (control group), 30% and 60% were fabricated. Then, evaluation of concrete compressive strength, evaluation of chloride ion diffusion coefficient and electrochemical impedance spectroscopy (EIS) were performed. As the GGBFS replacement ratio increased, the concrete compressive strength of the air cured specimens decreased compared to the underwater cured specimens. With respect to the chloride ion diffusion coefficient measurements, the coefficient decreased as the GGBFS replacement ratio increased. However, the diffusion coefficient of the air cured specimen relative to the underwater cured ones increased up to two times. The EIS results showed that as the GGBFS replacement ratio increased, |Z| increased in every frequency range. However, the |Z| of the air cured specimen was lower than the underwater cured one. This showed the same tendency as the evaluation results of the chloride ion diffusion coefficient.

## 1. Introduction

The performance of concrete is determined by its strength and durability. As the durability of concrete has recently emerged as a critical social issue, the development of high-quality concrete with high durability is urgently needed. The most important problem affecting the durability of reinforced concrete is the deterioration of embedded rebars due to corrosion [1,2]. The rebars embedded in concrete are protected from corrosion by stable oxide films that are formed on the surfaces in the strong alkaline (pH between 12.5 and 14) cement environment. However, the rebars are corroded by the reduced pH because of the neutralization of concrete or the penetration of chloride ions. The volume of the rebars thus increases because of the corrosion products resulting from the corrosion of rebars, which causes concrete cracks [3,4,5].

Reinforced concrete structures in areas affected by the marine environment require considerable maintenance costs owing to rebar corrosion caused by chloride ion penetration. The construction of reinforced concrete structures with excellent resistance to the marine environment is critical, not only from the point of view of stability of the structure, but also from the economic aspect [6,7,8]. One method to improve the durability of concrete against salt damage is to use additives such as blast furnace slag fine powder or fly ash, which are known to be effective at blocking intrusion by harmful elements such as chloride ions, by rendering a dense pore structures to concrete [9,10,11,12,13,14,15]. 

It has been reported in literature that using these additives in concrete is environmentally friendly because it helps reduce CO_2_ emissions into the atmosphere [16]. Furthermore, it is known that the additives considerably decrease the permeability and diffusion rate of water and chloride ions in hardened concrete, suppress the sulfate and alkali-aggregate reactions and prevent corrosion of the rebars [17,18,19,20,21,22,23,24,25,26,27,28,29,30].

However, one problem that has been continuously noted is that the replacement of cement with ground granulated blast furnace slag (GGBFS) is associated with high variations in quality and high dependency on the curing environment. In particular, with regard to the curing environment, it is reported that when cement is replaced with GGBFS, the pore structure of concrete changes greatly among underwater curing, air dry curing and carbonation [31,32,33,34,35,36,37]. Furthermore, it is reported that the formation of pores is closely related to the material curing environment and the pore size and distribution inside concrete are closely related to the penetration and diffusion of deteriorating factors outside the concrete [28,34,35,38,39,40,41].

Thus, when GGBFS is used in concrete, the curing conditions have a high correlation with resistance performance against the deteriorating factors of concrete. Therefore, systematic research is required on the variation of resistance of concrete with GGBFS to salt damage according to the curing conditions. To evaluate the effects of curing conditions on concrete containing GGBFS, in this study, the chloride ion penetration resistance of concrete was measured and compared for different curing environments for concrete specimens that were further divided as air-dry cured and underwater cured. Furthermore, the impedances of rebars embedded in concrete were measured and compared. The results of this study are expected to provide basic data on the effects of curing conditions on resistance to chloride ingress of concrete with GGBFS.

## 2. Materials and Specimens

### 2.1. Materials

Type 1 ordinary Portland cement (OPC) of ASTM C 150 [42] with a density of 3.15 g/cm^3^ (S Company, Seoul, South Korea) was used in this study. In addition, Grade-80 GGBFS of ASTM C 989 [43] with a Blaine fineness of 4000 cm^2^/g (S Company, Seoul, South Korea) was used. Table 1 lists the chemical compositions according to the binder.

To reduce the effect of aggregates between the rebar and concrete cover, the maximum size of coarse aggregates was limited to 13 mm and the fine aggregates were prepared by mixing crushed sand and washed sea sand. In South Korea, it is common to use a combination of crushed aggregates and washed (decontaminated chloride) sea sand owing to the depletion of river sand [44,45]. The superplasticizer and air-entering & high-range water reducing agent were used together to secure the fluidity and air contents of concrete. Also, to control the effects of chloride ion in chemical admixture, the admixture from S company (South Korea) were used, which are not contained chloride ions.

### 2.2. Concrete Mix Proportion

For this experiment, nine types of concrete specimens were prepared according to the GGBFS replacement ratio and W/B ratio: three GGBFS replacement ratios of OPC 100%, OPC 70% + GGBFS 30% and OPC 40% + GGBFS 60% and three W/B ratios of 40%, 50% and 60%. Table 2 lists the concrete mix proportions according to the experiment levels.

### 2.3. Specimens

#### 2.3.1. Specimen for Chloride Ion Migration Coefficient of Concrete 

**Figure 1:**
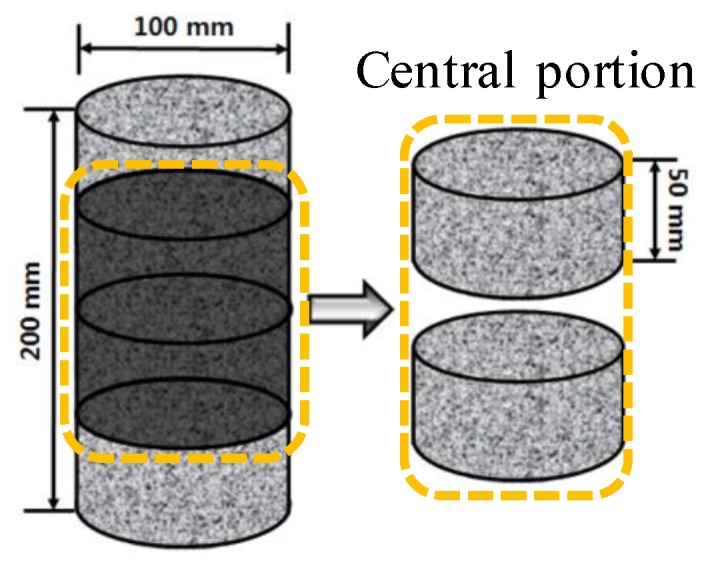
Schematic diagram of specimen for chloride penetration test.

Immediately after the concrete was mixed for specimen fabrication, it was poured into a Ø100 × 200 mm cylindrical mold and sealed. The specimen was demolded 24 h later and cured for 28 days. After the curing was complete, the specimen was cut into samples of height 50 mm and the two samples from the middle of the specimen were used. Figure 1 shows the schematic diagram of the concrete specimens for chloride penetration test [46].

#### 2.3.2. Specimen for Electrochemical Measurements

**Figure 2:**
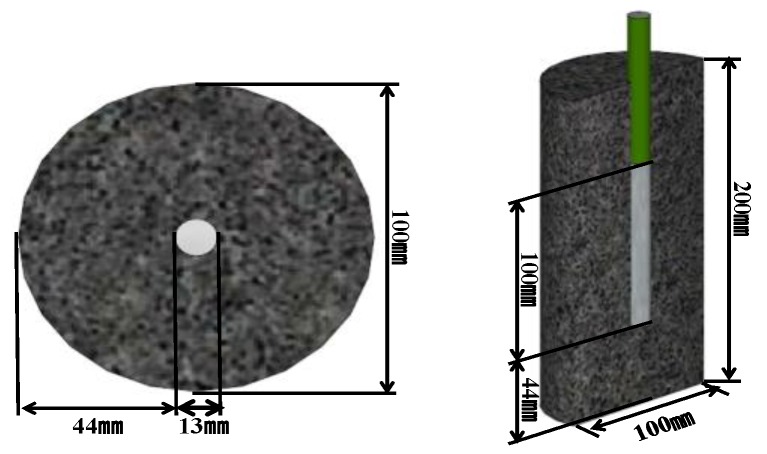
Schematic diagram of specimen for electrochemical measurement.

The reinforced concrete specimens were prepared by fixing a ∅13 mm rebar at the center of the Ø100 × 200 mm cylindrical mold and then pouring the concrete into the mold. For the rebar embedded in the concrete, SD-400 deformed rebars were used. After the rebar surface was cleaned by grit blast, an epoxy coating was applied to the surface and only a length of 100 mm at the center of the concrete specimen was exposed. Figure 2 shows a schematic of the fabricated reinforced concrete specimen.

#### 2.3.3. Curing Method

To compare the salt damage resistances of concrete specimens according to the curing conditions, the concrete specimen poured in the mold was demolded after 24 h. Then, the concrete specimens were cured separately for by air and underwater curing for 28 days. The air curing conditions were a temperature of 20 ± 2 °C and relative humidity of 60 ± 5%. For underwater curing, the specimen was completely immersed in water at a temperature of 20 ± 2 °C.

## 3. Experimental Method

### 3.1. Evaluation of Concrete Compressive Strength

The concrete compressive strength was evaluated in accordance with ASTM C39 [47] at 3, 7 and 28 days of curing. For each experimental level, nine cylindrical concrete specimens of Ø100 × 200 mm were fabricated. The concrete compressive strengths of three specimens were measured simultaneously and their average value was used.

### 3.2. Evaluation of Resistance to Chloride Ion Penetration of Concrete

**Figure 3:**
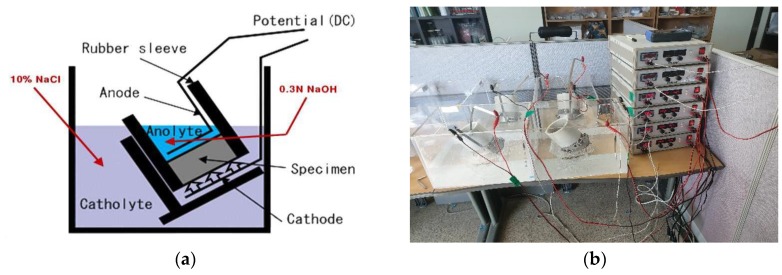
(**a**) One arrangement of the migration set-up; (**b**) Photograph image of NT BUILD 492 Test setup.

To evaluate resistance to chloride ingress of concrete, it is most accurate to conduct exposure test. However, exposure test need a very long time. Therefore, it is common to evaluate the resistance and diffusion coefficient of harmful ions of concrete using electrochemical acceleration. In general, ASTM C 1202 and NT-BUILD 492 are used for evaluating chloride ingress in concrete. In this study, among the chloride ion penetration resistance evaluation methods, the chloride migration coefficient from non-steady-state migration experiments were conducted by NT BUILD 492 [46], which is a northern European regulation often used as a quantitative evaluation method. Figure 3 shows the schematic of the cells configured for this experiment and photographs of the installed experimental setup.

**Figure 4:**
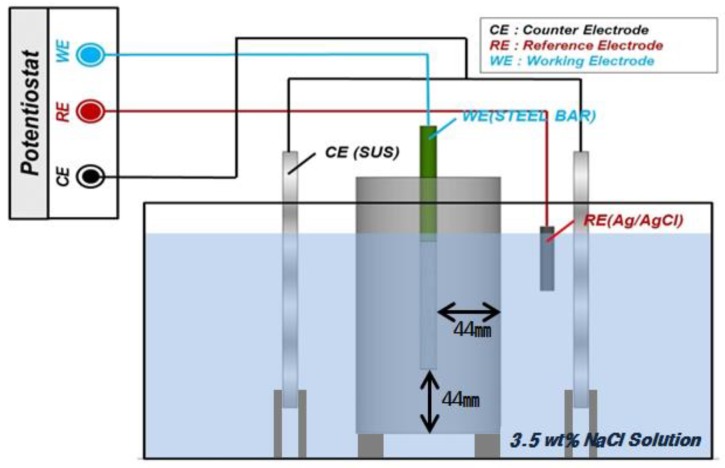
Schematic diagram of electrochemical impedance spectroscopy (EIS) experiment.

This experiment is a non-steady-state electric migration experiment to determine the chloride migration coefficient of the repair material composed of concrete, mortar and cement. Concrete specimens of size Ø100 × 50 mm were prepared, saturated Ca(OH)_2_ solution was filled in a desiccator using a vacuum pump and the specimen was immersed in the solution. The inside of the desiccator was maintained in a vacuum state using a vacuum pump and the pores in the concrete were filled with saturated Ca(OH)_2_ solution. When the pretreatment was complete, chloride ion diffusion cells were prepared as shown in Figure 4. The anode was filled with 0.3M NaOH and the cathode was filled with 10% NaOH solution. Then, the initial current value (l_30V_) was measured. Next, the actual applied voltage was adjusted by finding the range of current according to the initial value in Table 3. The chloride ion penetration resistance was tested using the potential difference by selecting an appropriate time according to the current.

After the test, the specimen was split in the axial direction into two pieces. When 0.1N AgNO_3_ solution was sprayed on the split section, discolored parts appeared on the specimen depending on the penetration depth of the chloride ions. The diffusion coefficient was determined using the average of seven measurements in 10 mm intervals of the chloride penetration depth.

Equation (1) is used to estimate the diffusion coefficient from the chloride ion penetration depth [46].

(1) $$D_{nssm}\;=\;\frac{0.0239\left(273+T\right)L}{\left(U-2\right)t}\left(x_{d}-0.0238\sqrt{\frac{\left(273+T\right)Lx_{d}}{U-2}}\right)$$

where

*D_nssm_* is a non-steady-state migration coefficient (×10^−12^ m^2^/s)
*U* is the absolute value of the applied voltage (V)
*T* is the average of the initial and final temperatures in the anolyte solution (°C)
*L* is the thickness of the specimen (mm)
$x_{d}$ is the average value of the penetration depths (mm)
*t* is the test duration (hour)

### 3.3. Electrochemical Measurement

The impedance of the embedded rebar based on the concrete mix and curing conditions was measured using electrochemical impedance spectroscopy (EIS). The experimental apparatus used in this experiment included a Potentiostat (PGSTAT302N, Metrohm Autolab, Kanaalweg, Utrecht, Netherlands). The three-electrode system for the experiment was setup using the rebar as the working electrode (WE), STS304 as the counter electrode (CE) and Ag/AgCl as the reference electrode (RE). Table 4 outlines the EIS experiment and Figure 4 shows a schematic of the cells configured for this experiment [48,49,50].

**Figure 5:**
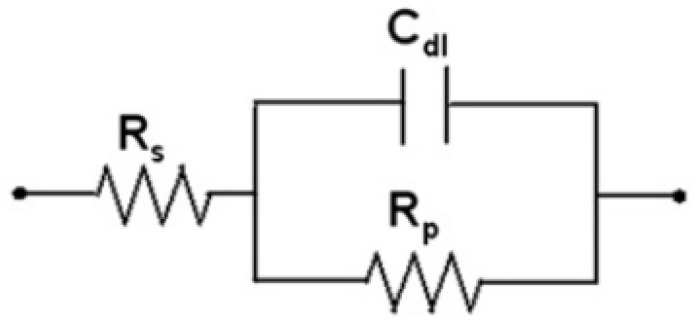
Schematic diagram of a simple electrochemical electrode system.

According to electrochemical theory, a simple electrochemical electrode system can be described by an equivalent circuit [48,49,50], as shown in Figure 5.

**Figure 6:**
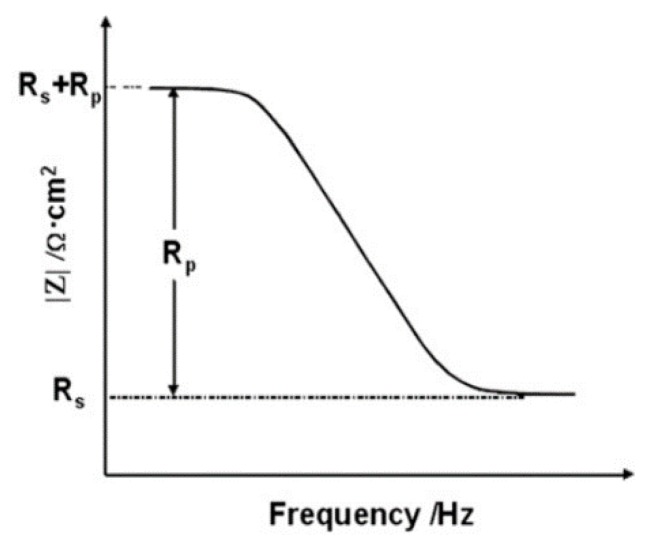
Bode modulus plot of EIS.

Here, R_s_ denotes the solution resistance, C_dl_ denotes the capacitance of an electrical double layer and R_p_ denotes the polarization resistance by charge transfer reaction. Figure 6 shows a Bode plot corresponding to the impedance result graphs for Figure 5.

R_s_ corresponds to the impedance of the highest frequency and R_s_ + R_p_ corresponds to the impedance of the lowest frequency. Regarding the rebars embedded in concrete, the R_p_ of the rebar is composed of the resistance of the film R_f_ and the charge transfer resistance R_ct_ [48,49,50,51,52,53]. Therefore, the corrosion resistance performance of the rebars can be represented by R_p_. In the EIS experiment, it can be assumed that the value of the Bode plot at 100 kHz is equal to R_s_ and the value of the plot at 0.1 Hz is equal to R_s_ + R_p_. Therefore, R_p_ can be calculated.

## 4. Results and Discussion

### 4.1. Result of Concrete Compressive Strength

**Figure 7:**
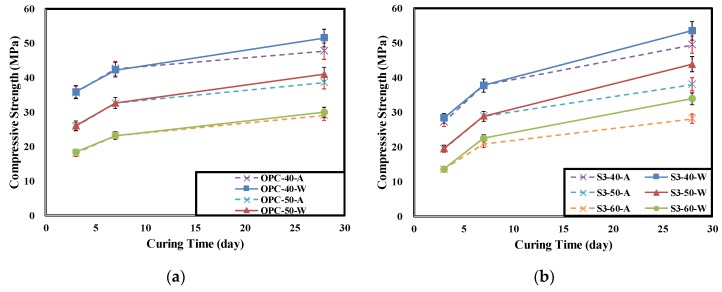

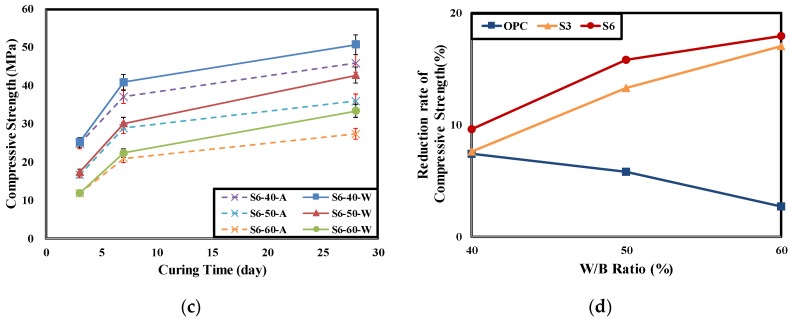
Compressive strength of concrete according to curing day and curing condition. (**a**) Ordinary Portland cement (OPC), (**b**) OPC 70% + GGBFS 30%, (**c**) OPC 40% + GGBFS 60%, (**d**) Reduction rate of Compressive strength according to W/B(28d).

Table 5 shows the concrete compressive strength for each experimental level according to the curing conditions and age. Figure 7 show the changes in concrete compressive strengths according to the curing conditions and age. 

Equation (2) is used for estimating the rate of change for compressive strength by curing condition.

(2) $$Rate\;of\;change\left(\%\right)=\;\frac{IA-UW}{UW}\times 100\;\left(\%\right)$$

where

*UW* is the result of underwater curing condition
*IA* is the result of air-dry curing condition

The descending order of the measured initial compressive strengths of the concretes is OPC > S3 > S6. However, the 28-day compressive strength of the S3-40-W specimen was 53.6 MPa, which is higher than the 51.7 MPa of the OPC-40-W specimen. The difference was less than 2 MPa, which is not large. This seems to be due to the expression of compressive strength caused by the latent hydraulic activity of the GGBFS. In every specimen, the tendency of decreasing compressive strength with increasing W/B ratio was observed [24,31,39].

In particular, the concrete mixed with GGBFS showed higher dependence on underwater curing for the strength expression than on the OPC. The strength reduction rate of the air-dry cured specimen relative to the underwater cured specimen was less than 8% for the OPC. However, the concrete mixed with additives showed 10–20% strength reduction rates at most levels. The OPC showed a lower strength reduction rate as the W/B increased. This seems to be because many hydration products were generated by the hydration reaction of cement as there was sufficient curing water. The specimen mixed with GGBFS showed a higher strength reduction rate as the W/B increased. This is attributed to the fact that the curing water was insufficient for latent hydraulic activity of the GGBFS and the generation of hydration products decreased owing to the lower absolute quantity of cement. Furthermore, the low age (28 d) was assumed to have had an effect as well. The S3-40 specimen showed a low strength reduction rate. This seems to be because the amount of mix water was sufficient for hydration of the cement and the GGBFS also partially showed latent hydraulic activity [24,39].

Furthermore, the concrete mixed with GGBFS that was air cured showed limited strength expression because the inside of the concrete was not dense owing to slow hydration reaction speed. This is also expected to affect the penetration resistance of chloride ions, which is affected by the porosity. Consequently, as the W/B is lower in the air curing condition, proper mixing of the GGBFS will be more effective in delaying rebar corrosion due to salt damage [24,39].

### 4.2. Results of Chloride Ion Diffusion Coefficient of Concrete

**Figure 8:**
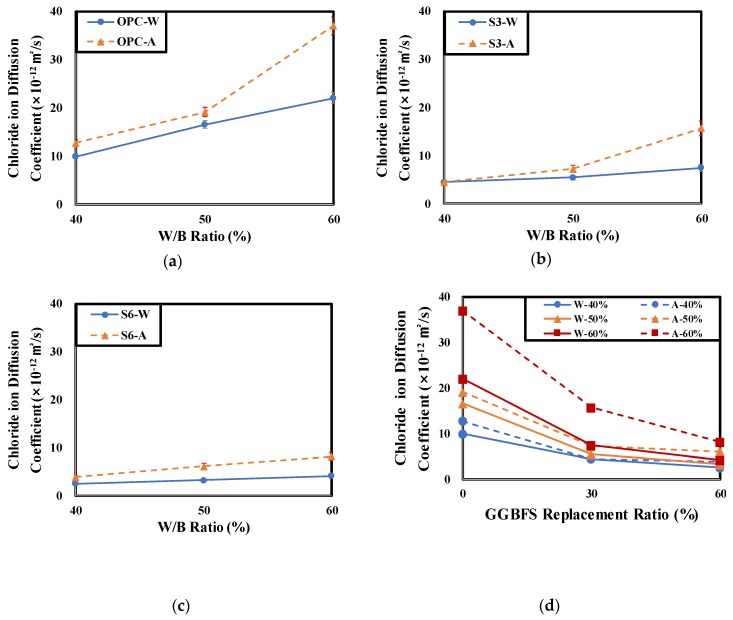
Chloride ion diffusion coefficient of concrete according to curing condition and W/B ratio. (**a**) OPC, (**b**) OPC 70% + GGBFS 30%, (**c**) OPC 40% + GGBFS 60%, (**d**) Chloride ion diffusion coefficient of all specimens according to GGBFS replacement ratio.

Table 6 and Figure 8 show the chloride ion diffusion coefficient changes of concrete at each experimental level according to the curing conditions and W/B ratio.

As a result of NT BUILD 492 test, Chloride ion diffusion coefficient increase according to W/B ratio increase. All 28-days air-dry cured specimens show higher chloride ion diffusion coefficient than all 28-days under-water cured specimens. Also, Increase rate of chloride ion diffusion coefficient of air-cured specimens by W/B are higher than one of water-cured specimens. Only the S3-40 specimen showed no change in the concrete chloride diffusion coefficient according to curing condition under the condition of W/B 40%. This seems to be because under the air curing condition at GGBFS and W/B replacements of 30% and 40%, dense pore structures were formed by the hydration of concrete and the latent hydraulic activity of GGBFS. In addition, at the W/B of 50% and 60% conditions, the chloride ion diffusion coefficient of the air cured specimen was higher than that of the underwater cured specimen. It was found that as the W/B increased, the chloride diffusion coefficient of the concrete according to the curing conditions increased [39].

In the case of the OPC specimens, the increase rate of the concrete chloride diffusion coefficient decreased by the curing condition as the W/B increased. However, in the case of the OPC with GGBFS specimens, the increase rate of the concrete chloride diffusion by curing condition increased as the W/B increased. The increase rate of OPC was lowest at 17.94% but the increase by absolute value was the highest. The specimen that showed the highest increase rate was S3. The increase rate of the chloride diffusion coefficient of S3 was the lowest at 40% W/B but it was 111% at 60% W/B. All specimens using GGBFS showed high increase rates of chloride diffusion coefficient by the curing conditions as the W/B increased. This is consistent with the tendency of decreasing compressive strength of the air cured concrete relative to the underwater cured concrete mentioned in Section 4.1. This is attributed to the fact that the pore water was not sufficient for the smooth progress of latent hydraulic activity of GGBFS in the air cured condition. Therefore, to secure salt damage resistance of concrete produced by replacing cement with GGBFS, special attention should be paid to the concrete curing condition [54].

### 4.3. Result of Electrochemical Measurements

**Figure 9:**
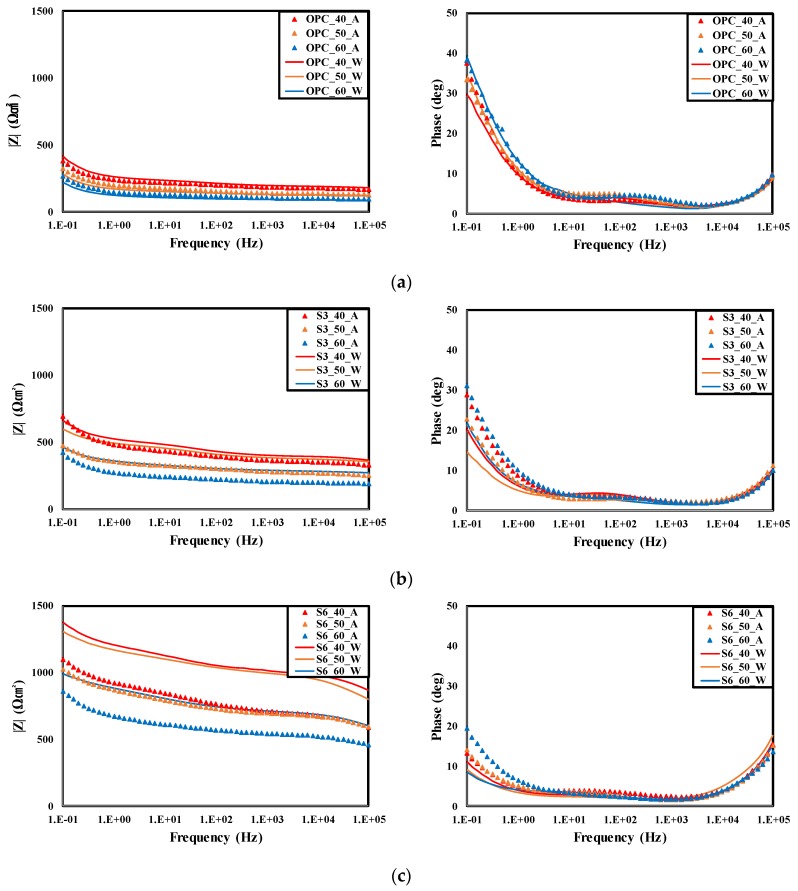
Bode modulus plot and phase plot of rebars embedded in concrete according to the curing condition. (**a**) OPC, (**b**) OPC 70% + GGBFS 30%, (**c**) OPC 40% + GGBFS 60%.

Figure 9 show the Bode Modulus and phase plots for EIS measurement results of rebars embedded in concrete according to the curing conditions.

The descending order of measured impedance |Z| of the rebars embedded in concrete according to the applied frequency range was S6 > S3 > OPC. As the GGBFS replacement ratio of cement increased, the impedances of the rebars embedded in concrete increased [23,52]. For the S6-40-W specimen, which showed the highest |Z|, the |Z| in the low-frequency range was 1374.26 Ωcm^2^, which is approximately 3.28 times that of the OPC-40-W specimen, 418.46 Ωcm^2^ and 2.06 times that of the |Z| of the S3-40-W specimen, 668.19 Ωcm^2^.

**Figure 10:**
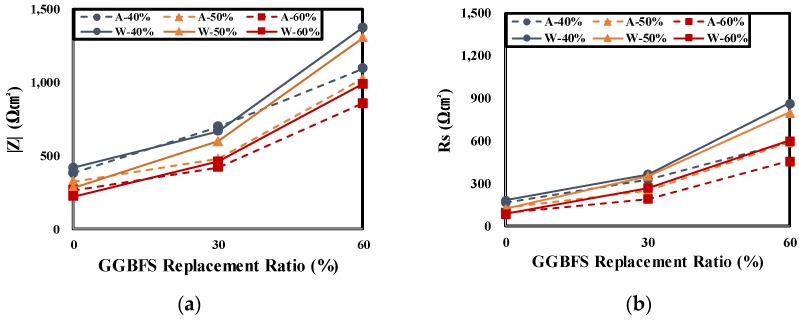

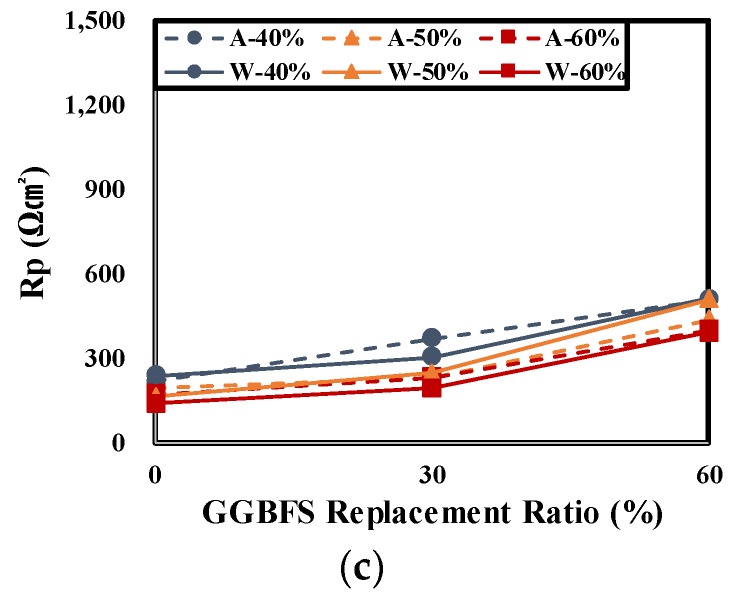
Impedance of rebars embedded in concrete according to the curing condition and GGBFS replacement ratio. (**a**) |Z| (Z_total_), (**b**) R_s_ (Z_min_) (**c**) R_p_ (Z_max_ − Z_min_).

Table 7 and Figure 10 show the R_s_ and R_p_ for EIS measurement results of rebars embedded in concrete according to the curing conditions and GGBFS replacement ratios.

The OPC specimens showed differences in |Z| depending on W/B and curing conditions but the differences were not large over the entire frequency range. In the case of the OPC specimen, the |Z| values of the rebars embedded in the air cured concrete and underwater cured concrete did not show differences. This suggests that the corrosion resistance performance of the air curing condition is not much different from that of the underwater curing condition. However, the lowest |Z| was observed at every experimental level and the |Z| was low at the low- and high-frequency ranges. This suggests that the |Z|(R_s_) in the high-frequency range decreased due to low chloride ion penetration resistance of the concrete of the OPC specimen. This also affects the |Z|(R_s_ + R_p_) in the low-frequency range and shows the same tendency as the measurement results of the concrete chloride diffusion coefficient in the previous experiment [53,54].

However, as the GGBFS replacement ratio increased and W/B decreased, the impedance of the rebars embedded in the air cured specimen decreased compared to the underwater cured specimen. In the case of the S6-40 specimen, the impedance |Z| of the low-frequency range was 1374.26 Ωcm^2^ for the underwater cured condition and 1095.78 Ωcm^2^ for the air cured condition. Thus, the |Z| decreased by 20.26% by the curing condition. Furthermore, the impedance |Z| of the high-frequency range was 864.38 Ωcm^2^ for the underwater cured condition and 588.41 Ωcm^2^ for the air cured condition. Thus, the |Z| decreased by 31.93% by the curing condition. It is considered that the increase in the chloride ion penetration resistance of concrete caused by the increase in the GGBFS replacement ratio greatly increases the |Z|(R_s_) in the high-frequency range, which also affects the |Z| in the low-frequency range. Furthermore, the increase of |Z| of the underwater cured specimen relative to the air cured specimen seems to be due to the dense pore structure of concrete resulting from insufficient pore water for latent hydraulic activity of the GGBFS. As the W/B increases, the amount of pore water increases and the difference of |Z| according to the curing condition decreases. Therefore, this shows the same tendency as that of the chloride diffusion coefficient measurement results of concrete.

**Figure 11:**
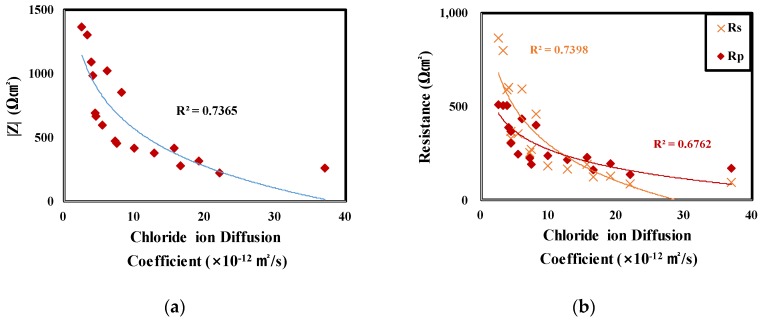
Relationship between impedance and chloride ion diffusion coefficient. (**a**) |Z| (Z_total_), (**b**) R_s_ (Z_min_) and R_p_ (Z_max_ − Z_min_).

Figure 11 shows the relationship between impedance and chloride ion diffusion coefficient.

As the chloride ion diffusion coefficient increases, it was confirmed that the impedance decreased. Results of comparing and evaluating R_s_ and R_p_ confirmed that the change of R_s_ in |Z| is larger than the change of R_p._ This is due to the decrease in the chloride ion diffusion coefficient that causes an increase of the solution resistance (R_s_) of the concrete. By measuring the impedances of the rebars embedded in concrete, the chloride ion diffusion coefficient of concrete can be determined [55,56]. This relationship is believed to be due to the difference in concrete pore structure according to curing conditions [31,54,55].

## 5. Conclusions

In this study, the effect of curing conditions on resistance to chloride ingress in concrete using GGBFS was investigated experimentally. Curing conditions were classified into air and underwater curing and cement type of concrete was classified as 0%, 30% and 60% according to the GGBFS replacement ratio. Resistance to chloride ingress was evaluated by measuring the chloride ion diffusion coefficient and impedance. The results are as follows.

1. The evaluation results of the concrete compressive strength showed that the compressive strength of the air cured specimen decreased compared to the underwater cured specimen. As the GGBFS replacement ratio increased, the compressive strength reduction rate increased. The reason for this seems to be that when air curing is performed, pore water is not enough for sufficient exhibition of latent hydraulic activity of the GGBFS and thus, it decreases the generation of hydration products.
2. The evaluation result of the concrete chloride ion diffusion coefficient showed that the chloride ion diffusion coefficient of the air cured specimen was higher than that of the underwater cured specimen. Furthermore, the higher the GGBFS replacement ratio, the lower is the chloride ion diffusion coefficient. The high fineness and latent hydraulic activity reaction of GGBFS decreased the concrete chloride ion diffusion coefficient compared to the OPC but when air curing was performed, the chloride ion diffusion coefficient increased relative to underwater curing.
3. The impedance measurement results of rebars embedded in concrete showed that the |Z| of the air cured specimen decreased in every frequency range relative to the underwater cured one at all levels. As the GGBFS replacement ratio increased, |Z| increased in every frequency range. This showed the same tendency as the evaluation result of the concrete chloride ion diffusion coefficient. It is considered that the higher the chloride penetration resistance is, the higher is the |Z| value of the rebars embedded in concrete.
4. As the GGBFS replacement rate increased, the performance of the underwater cured specimen was higher relative to the air cured specimen in every experiment. This suggests a high dependence on underwater curing. Therefore, when concrete is manufactured using GGBFS, special care should be devoted to the curing conditions.

## Figures and Tables

**Table 1:** Chemical compositions of cement and ground granulated blast furnace slag (GGBFS).

Name	Chemical Compositions (%)									
	SiO_2_	Al_2_O_3_	TiO_2_	Fe_2_O_3_	CaO	MgO	SO_3_	K_2_O	etc.	L.O.I
OPC	19.74	5.33	0.30	2.93	61.74	3.78	2.47	0.89	2.82	2.3
GGBFS	33.35	13.36	0.59	0.33	44.62	4.12	2.69	0.41	0.53	0.1

**Table 2:** Concrete mix proportion.

Name	W/B (%)	Unit Weight (kg/m^3^)						Unit Weight (g/m^3^)	
		W	C	GG BFS	S1 ^1^	S2 ^2^	G ^3^	S.P.	A.E.
OPC-40	40	180	450	-	553	240	867	3600	150
OPC-50	50	180	360	-	607	260	867	3240	117
OPC-60	60	180	300	-	640	273	867	3007	93
S3-40	40	180	315	135	547	240	867	3150	157
S3-50	50	180	252	108	600	260	867	2880	130
S3-60	60	180	210	90	640	273	867	2400	120
S6-40	40	180	180	270	540	233	867	2700	430
S6-50	50	180	144	216	600	253	867	2160	300
S6-60	60	180	120	180	633	267	867	2100	250

^1^ Crushed Sand, ^2^ Sea Sand (Washed), ^3^ Maximum size of coarse aggregate: 13 mm.

**Table 3:** Test voltage and duration for concrete specimen with normal binder content [46].

Initial Current I_30V_ (with 30V) (mA)	Applied Voltage U (after Adjustment) (V)	Possible New Initial Current I_0_ (mA)	Test Duration (Hour)
I_0_ < 5	60	I_0_ < 10	96
5 ≤ I_0_ < 10	60	10 ≤ I_0_ < 20	48
10 ≤ I_0_ < 15	60	20 ≤ I_0_ < 30	24
15 ≤ I_0_ < 20	50	25 ≤ I_0_ < 35	24
20 ≤ I_0_ < 30	40	25 ≤ I_0_ < 40	24
30 ≤ I_0_ < 40	35	35 ≤ I_0_ < 50	24
40 ≤ I_0_ < 60	30	40 ≤ I_0_ < 60	24
60 ≤ I_0_ < 90	25	50 ≤ I_0_ < 75	24
90 ≤ I_0_ < 120	20	60 ≤ I_0_ < 80	24
120 ≤ I_0_ < 180	15	60 ≤ I_0_ < 90	24
180 ≤ I_0_ < 360	10	60 ≤ I_0_ < 120	24
I_0_ ≥ 360	10	I_0_ ≥ 120	6

**Table 4:** Experimental overview of EIS experiment.

Frequency Range	10^5^ ~ 10^−2^ Hz
Specimen Size	Ø100 × 200 mm^2^
Cover concrete	44 mm
WE	Ø13 mm rebar (SD400)
RE	Ag/AgCl
CE	STS 304

**Table 5:** Result of compressive strength test.

Name		Compressive Strength (MPa)			Rate of Change 28d (%)
		3d	7d	28d	
OPC-40	W *	35.9	42.3	51.7	−7.43
	A **	35.8	42.7	47.8	
OPC-50	W	26.1	32.7	41.1	−5.79
	A	25.9	32.7	38.7	
OPC-60	W	18.4	23.2	30.0	−2.66
	A	18.0	23.2	29.2	
S3-40	W	28.3	37.8	53.6	−7.58
	A	27.2	37.8	49.5	
S3-50	W	19.6	28.9	43.9	−13.28
	A	19.4	28.9	38.1	
S3-60	W	13.6	22.6	34.0	−17.06
	A	13.6	20.9	28.2	
S6-40	W	25.2	40.8	50.7	−9.61
	A	24.7	37.1	45.8	
S6-50	W	17.3	30.1	42.7	−15.85
	A	16.6	28.8	35.9	
S6-60	W	11.8	22.4	33.4	−17.98
	A	11.8	20.9	27.4	

**Table 6:** Result of Chloride diffusion coefficient of concrete according to curing condition.

Name		Chloride Diffusion Coefficient (28d) (×10^−12^ m^2^/s)	Rate of Change (%)
OPC-40	W	9.92	28.53
	A	12.75	
OPC-50	W	16.56	15.70
	A	19.16	
OPC-60	W	22.05	17.94
	A	37.03	
S3-40	W	4.47	−0.45
	A	4.45	
S3-50	W	5.41	33.83
	A	7.24	
S3-60	W	7.42	111.19
	A	15.67	
S6-40	W	2.51	52.99
	A	3.84	
S6-50	W	3.24	87.96
	A	6.09	
S6-60	W	4.12	96.60
	A	8.10	

**Table 7:** Result of EIS according to curing condition.

Name		|Z|_max_ (Ωcm^2^)	|Z|_min_ (R_s_) (Ωcm^2^)	|Z|_max_ − |Z|_min_ (R_p_) (Ωcm^2^)
OPC-40	W	418.463	181.009	237.454
	A	382.322	166.431	215.891
OPC-50	W	281.896	121.460	160.436
	A	323.150	127.378	195.772
OPC-60	W	223.554	87.651	135.903
	A	263.696	94.007	169.689
S3-40	W	668.187	365.856	302.331
	A	697.065	329.151	367.914
S3-50	W	598.828	353.875	244.953
	A	478.571	252.401	226.170
S3-60	W	460.889	270.274	190.615
	A	421.707	191.777	229.930
S6-40	W	1374.260	864.382	509.878
	A	1095.780	588.412	507.368
S6-50	W	1306.230	800.663	505.567
	A	1027.270	593.435	433.835
S6-60	W	990.183	601.283	388.900
	A	857.888	458.342	399.546
